# Soil organic amendments with *Polygonum cuspidatum* residues enhance growth, leaf gas exchange, and bioactive component levels

**DOI:** 10.3389/fpls.2025.1594905

**Published:** 2025-04-25

**Authors:** Lei Liu, Hong-Na Mu, Ze-Zhi Zhang

**Affiliations:** ^1^ College of Architecture and Design, Yangtze University College of Arts and Sciences, Jingzhou, China; ^2^ College of Horticulture and Gardening, Yangtze University, Jingzhou, China; ^3^ Shiyan Academy of Agricultural Sciences, Shiyan, China

**Keywords:** compound fertilizer, indole-3-acetic acid, medicinal plant, polydatin, resveratrol

## Abstract

The extracted residue of *Polygonum cuspidatum* (a valuable medicinal plant) rhizome is discarded as waste, while it is unclear whether returning this residue to the field would be beneficial for the growth and its active component production of *P*. *cuspidatum*. This study aimed to investigate the effects of applying *P*. *cuspidatum* residues (PRs) to the field on plant growth, photosynthetic activities, root indole-3-acetic acid (IAA) and zeatin riboside (ZR) levels, active component (polydatin, resveratrol, and emodin) contents, and the expression of resveratrol-associated genes (*PcRS* and *PcPKS1*) in *P*. *cuspidatum* plants. The experiment comprised four treatments, namely, the application of potassium sulfate compound fertilizer at a rate of 50 kg/667 m^2^ and the application of PRs at rates of 1500 kg/667 m^2^ (PR1500), 2500 kg/667 m^2^ (PR2500), and 4000 kg/667 m^2^ (PR4000), along with a control (CK) receiving no additional substances. Two years later, the application of both the compound fertilizer and PR treatments led to substantial increases in plant height, stem diameter, leaf number, number of nodes on main stems, and aboveground (leaf, branch, and main stem) and root biomass production, depending on used doses of PRs applied. Among them, the PR2500 treatment exhibited the superior performance. Additionally, these treatments significantly boosted root IAA (11.0−41.7%) and ZR (17.8−46.0%) levels, with the PR2500 treatment demonstrating the highest efficacy. Root IAA and ZR levels were significantly (*p* < 0.01) positively correlated with root biomass. All treatments, except for PR4000, significantly elevated SPAD values, net photosynthesis rate, transpiration rate, and intercellular CO_2_ concentration in leaves, with PR2500 showing the most pronounced improvements. Fertilization and PR treatments significantly boosted root polydatin (6.6−22.0%), emodin (12.1−43.3%), and resveratrol (17.8−69.3%, except for PR4000) levels, along with a significant up-regulation of *PcRS* expression and a significant down-regulation of *PcPKS1* expression in roots. In short, organic amendments like PRs, particularly at a rate of 2500 kg/667 m^2^, can be a viable alternative to traditional fertilizers for enhancing the plant growth and its active component levels of *P*. *cuspidatum*, making them a cornerstone of eco-friendly farming practices and sustainable agriculture.

## Introduction

*Polygonum cuspidatum*, a perennial shrub-like herbaceous medicinal plant, is widely distributed across East Asia, covering China, Japan, and the Korean Peninsula (Ye et al., 2021; Sun et al., 2022). In China, it is predominantly found in the eastern, central, southern, and southwestern regions (Chen et al., 2020). It exhibits strong environmental adaptability, capable of growing in various soil types. The plants contain several pharmacologically active chemical compounds, including: (i) anthraquinone derivatives such as emodin, chrysophanol, and physcion, which exhibit antibacterial, anti-inflammatory, and antioxidant properties; (ii) stilbenes, like resveratrol, known for their antioxidant, anticancer, and anti-inflammatory effects; (iii) flavonoids, including quercetin and kaempferol, which possess antioxidant, anti-inflammatory, and immunomodulatory activities; and (iv) polysaccharides, demonstrating immunomodulatory and antitumor properties (Zhou et al., 2003; Shan et al., 2008; Zhang et al., 2013; Lee et al., 2015; Ke et al., 2023; Lai et al., 2024). *P*. *cuspidatum* extracts, particularly for resveratrol, are widely used in dietary supplements, but they lack formal GRAS (Generally recognized as safe) status from the US FDA (https://www.fda.gov/food/gras-notice-inventory). Regulatory agencies such as EFSA (https://www.efsa.europa.eu/) and WHO (https://www.fao.org/food/food-safety-quality/scientific-advice/jecfa/en/) have not established a specific acceptable daily intake for *P. cuspidatum*; however, resveratrol doses up to 1500 mg/day have been used in clinical studies without significant adverse effects based on EFSA. Residual components in *P*. *cuspidatum* possess certain pharmacological and physiological properties (Tian et al., 2012).

The escalating demand for the medicinal components of *P*. *cuspidatum* has led to a substantial increase in its cultivated area and production. After the extraction of active chemical compounds, the resulting *P*. *cuspidatum* residues (PRs) are typically treated as waste. Nevertheless, PRs still contain nutritional components (e.g., fiber, residual sugars, and minerals), chemical compounds (e.g., various secondary metabolites and other bioactive substances that were not fully extracted), and cellular materials (plant cell walls and other structural components) (Zhang and Liu, 2015; Zhou et al., 2018). As a result, recognizing PRs as a potential organic resource rather than just waste is crucial for the sustainable and economically viable processing of *P*. *cuspidatum*. PRs hold considerable potential for further utilization (Zhou et al., 2018). For instance, employing *P*. *cuspidatum* residues as a substitute for traditional substrates in the cultivation of *Pleurotus eryngii* not only mitigates the environmental and air pollution caused by the indiscriminate disposal and incineration of the residues but also reduces the production costs of the mushrooms, thereby enhancing overall economic efficiency (Chang et al., 2019; Sun et al., 2021). However, this effect was dependent on the proportion of *P*. *cuspidatum* residues used, with the most prominent effect achieved by a treatment of 20% *P*. *cuspidatum* residues in the cultivation substrate (Chang et al., 2019).

Organic amendments, such as compost, manure, biochar, and plant residues, are integral to sustainable agriculture (Matisic et al., 2024; Xu et al., 2024). They contribute to enhancing soil health, improving crop productivity, and reducing environmental impacts. Organic amendments serve as a food source for soil microorganisms, promoting microbial diversity and activity (Shu et al., 2022). This, in turn, enhances nutrient cycling, organic matter decomposition, and the production of plant growth-promoting substances (Chaudhari et al., 2021; Liang et al., 2025). In fact, *P*. *cuspidatum* residues are rich in cellulose and various mineral elements such as nitrogen, phosphorus, and potassium (Zhang and Liu, 2015), suggesting their potential as a soil amendment and organic fertilizer. Incorporating *P*. *cuspidatum* residues into the soil is reasonable to expect improvements in soil structure, increased soil fertility, and promotion of plant growth.

Nevertheless, there is no direct evidence indicating that returning the residue of *P*. *cuspidatum* rhizomes after active ingredient extraction to the field has significant benefits for the plant growth. If PRs can enhance the growth and bioactive compound production of *P*. *cuspidatum* plants, this approach would simultaneously reduce fertilizer usage and lower the costly waste disposal expenses and environmental risks associated with PRs. The objective of this study was to analyze the effects of *P*. *cuspidatum* residues returning to field on the growth, active compound (polydatin, resveratrol, and emodin) levels, and soil structure of *P*. *cuspidatum* plants, so as to provide technical guidance for the subsequent comprehensive utilization of *P*. *cuspidatum* residues.

## Materials and methods

### Plant culture and experimental design

The experiment was initiated on March 10, 2022, in Zhujiawan (110.713140°E, 32.057560°N), Hongta, Fang County, Hubei Province, China. The experimental site features a subtropical monsoon climate, with an annual sunshine duration of 1700 to 2000 hours, an average temperature of 10°C to 15°C, and an annual precipitation of 750 mm to 1160 mm. The soil at the site has a pH of 6.4, an alkaline hydrolyzable nitrogen content of 103.7 mg/kg, an Olsen-P of 12.08 mg/kg, and an organic matter content of 13.54 mg/g.

The cultivation method employed was ridge tillage with furrows, where the furrow depth was 15−20 cm, the furrow width was 30 cm, the bed width was 100 cm, and the bed length was 40 m. The planting configuration on the bed surface was double rows with a plant spacing of 30×40 cm. The tubers of *P*. *cuspidatum*, each weighing 80−105 g and having at least 2 buds, were provided by Shiyan Academy of Agricultural Sciences, Shiyan, China.

After extracting emodin, polydatin, and resveratrol from the 3-year-old *P*. *cuspidatum* rhizomes, the residue was deposited and sun-dried to obtain *P*. *cuspidatum* residues. The following treatments were applied into the soil during tuber transplantation: (i) control (CK) with no additional substance application; (ii) application of potassium sulfate compound fertilizer (N: P_2_O_5_: K_2_O = 14: 16: 15, total nutrients ≥45%) at a rate of 50 kg/667 m^2^ (compound fertilizer); (iii) application of *P*. *cuspidatum* residues (PRs) at a rate of 1500 kg/667 m^2^ (PR1500; 22.5 tons per hectare); and (iv) application of PRs at a rate of 2500 kg/667 m^2^ (PR2500; 37.5 tons per hectare); (v) application of PRs at a rate of 4000 kg/667 m^2^ (PR4000; 60 tons per hectare). The application rate of *P*. *cuspidatum* residue in this study was selected based on typical doses of plant residues used in agricultural practices (5–30 tons per hectare) (Lehmann et al., 2011; Chaudhari et al., 2021), as well as the dosage commonly applied by local cultivators in Shiyan, Hubei, China. The potassium sulfate compound fertilizer was provided by Xinyangfeng Agricultural Technology Co., Ltd. (Jingmen, China). The experiment was arranged in a randomized complete block design with five treatments, each replicated five times, with each replicate covering an area of 3 m^2^. Subsequently, the plants were managed in the field according to the grower’s regular practices. Such field management was maintained until July 2024, after which analyses of plant growth, leaf physiological activity, and root sample collection were carried out.

### Determination of plant growth variables and leaf gas exchange

Prior to plant harvesting, the plant height and stem diameter (10 cm above the ground) were directly measured using a measuring tape (DL9005B, Ningbo, China). Additionally, the number of stem nodes and leaves were counted manually. Furthermore, starting at 9:00 AM on a clear day before harvest, gas exchange analysis was conducted on the top third leaf using a LI-6400 portable photosynthesis system (LI-COR, Lincoln, USA), equipped with a buffer bottle to control CO_2_ concentration and a 6400-02B LED red/blue light source chamber. Data including net photosynthetic rate, transpiration rate, stomatal conductance, and intercellular CO_2_ concentration, was recorded after the system readings stabilized. Leaf chlorophyll values were measured using a portable Soil-Plant analysis development (SPAD) chlorophyll meter (502Plus, Osaka, Japan). On July 15, 2024, the plants were divided into leaves, main stems, lateral branches, and roots, and each part was weighed separately.

### Determination of indole-3-acetic acid and zeatin riboside levels in roots

The levels of endogenous IAA and ZR in the roots were determined using the enzyme-linked immunosorbent assay (ELISA). Approximately 0.5 g of fresh root samples was homogenized in 5 mL of 80% methanol solutions in an ice bath. The extraction was carried out at 4°C for 4 h. The homogenate was then centrifuged at 1,000×g/min for 15 min, and the supernatant was collected. The supernatant was passed through a C-18 column, and the eluent was dried under nitrogen at 45°C. The dried samples were then assayed using the ELISA kits for plant IAA (JM-01121P2) and ZR (JM-01037P2), respectively, provided by Jiangsu Jingmei Biotechnology Co., LTD (Yancheng, China), following the user manual instructions.

### Determination of active component levels in roots

The analysis of active component levels in roots was conducted following the method described by Yu et al. (2016). Briefly, 0.2 g of dried root powder was extracted with 25 mL of 60% ethanol under ultrasonication (50 kHz) for 30 min. The supernatant was collected after filtration and further passed through a 0.22 μm microporous membrane to obtain the sample solution. The chromatographic conditions were as follows: an Zorbax XDB-C18 column (4.6 × 250 mm, 5 μm; Agilent, Pal Alto, USA) was used; the mobile phase consisted of acetonitrile-0.5% acetic acid in water; the elution gradient was as follows: 0−10 min, 15%; 10−18 min, 15%−25%; 18−35 min, 25%−35%; 35−48 min, 35%−70%; 48−65 min, 70%−78%. The flow rate was set at 1.0 mL/min; the column temperature was maintained at 30°C; the detection wavelength was 290 nm; and the injection volume was 10 μL.

### Determination of gene expression levels in roots

The RNA from the roots was isolated using the Quick RNA Extraction Kit (Huayueyang, Beijing, China), followed by its conversion into cDNA. A *resveratrol synthase* gene (*PcRS*; DQ900615.1) and a *three-intron type III polyketide synthase 1 (PcPKS1*; EF090604.1) (Ma et al., 2009; Deng et al., 2022) were chosen, and specific primer sequences (**Supplementary Table S1**) were designed using Primer Premier 5.0. The qRT-PCR reactions were carried out, with β-actin serving as the reference gene and five biological replicates. The gene expression levels were quantified using the 2^-ΔΔCt^ method (Livak and Schmittgen, 2001), and the results were standardized against those of the CK treatment.

### Data analysis

Statistical analyses were performed using SAS software. One-way analysis of variance and Duncan’s multiple range test (*p* < 0.05) were used to conduct variance analysis and significant differences for the experimental data. Pearson’s correlation coefficients were presented to assess the relationships between variables. All measurements were replicated five times.

## Results

### Changes in plant growth performance

The application of fertilizer and PR treatments both significantly enhanced all plant growth measurements in comparison to CK (**Table 1**). PR at 2500 kg/667 m2 was significantly increased plant height (287 cm) and was different compared to CK and the other treatments; at the same times, PR4000 decreased plant height (228 cm) compared to PR2500. The minimum plant height (191 cm) was recorded at the CK treatment. PR2500 and PR4000 were significantly different in contrast to PR2500 and PR4000 in terms of stem diameters, leaf numbers, and node numbers on the main stem. The maximum stem diameters (31.97 and 31 mm), leaf numbers (838 and 811), and node numbers on the main stem (34.2 and 32.6) were achieved at both PR2500 and PR4000, respectively. Whereas CK showed the minimum stem diameter (18.81 mm), leaf number (495), and node number on the main stem (26.8 cm).

**Table 1:** Effects of *Polygonum cuspidatum* residues returning to field for sixteen months on the growth performance in three-year-old *P*. *cuspidatum* plants.

Treatments	Plant Height (cm)	Stem Diameter (mm)	Leaf Number	Number of nodes on Main Stems (num./plant)
CK	191 ± 12 d	18.81 ± 2.71 c	495 ± 153 c	26.8 ± 1.1 d
Compound fertilizer	239 ± 15 c	25.85 ± 3.49 b	672 ± 30 b	31.4 ± 2.1 bc
PR1500	266 ± 23 b	23.08 ± 1.21 bc	626 ± 18 b	29.8 ± 0.84 c
PR2500	287± 12 a	31.97 ± 5.86 a	838 ± 66 a	34.2 ± 2.59 a
PR4000	228 ± 9 c	31.00 ± 3.51 a	811 ± 61 a	32.6 ± 2.30 ab

Different letters following the data (means ± standard error, *n* = 5) from the same column indicated significant (*p* < 0.05) differences by Duncan’s tests. CK, no any additional treatment; compound fertilizer, application of potassium sulfate compound fertilizer; PR1500, application of *P*. *cuspidatum* residues at a rate of 1500 kg/667 m^2^; PR2500, application of *P*. *cuspidatum* residues at a rate of 2500 kg/667 m^2^; PR4000, application of *P*. *cuspidatum* residues at a rate of 4000 kg/667 m^2^.

Compared with the CK treatment, the PR treatments significantly enhanced biomass production in various plant tissues, including leaf, branch, main stem, leaf + branch + main stem, and root (**Table 2**). The biomass of leaf, branch, main stem, leaf + branch + main stem, and root was notably elevated by PR treatments, with increases of 49.1%, 133.3%, 160.0%, 94.7%, and 14.0%, respectively, under PR1500 treatment, with increases of 102.8%, 231.8%, 406.0%, 209.1%, and 24.1%, respectively, under PR2500 treatment, and with increases of 41.4%, 95.5%, 146.0%, 79.8%, and 11.8%, respectively, under PR4000 treatment. Overall, the PR2500 treatment demonstrated the most significant growth-promoting effects, surpassing the fertilizer treatment in promoting biomass production across all measured plant growth variables.

**Table 2:** Effects of *Polygonum cuspidatum* residues returning to field for two years on biomass production in three-year-old *P*. *cuspidatum* plants.

Treatments	Aboveground biomass (g/plant)				Root biomass (g/plant)
	Leaf	Branch	Main stem	Total	
CK	199 ± 12 c	66 ± 11 c	100 ± 12 c	365 ± 27 c	774 ± 41 d
Compound fertilizer	306 ± 62 b	150 ± 36 b	248 ± 37 b	704 ± 91 b	910 ± 24 b
PR1500	297 ± 20 b	154 ± 11 b	260 ± 10 b	711 ± 31 b	882 ± 13 bc
PR2500	404 ± 66 a	219 ± 21 a	506 ± 48 a	1129 ± 101 a	960 ± 11 a
PR4000	282 ± 22 b	129 ± 20 b	246 ± 27 b	657 ± 41 b	865 ± 11 c

Different letters following the data (means ± standard error, *n* = 5) from the same column indicated significant (*p* < 0.05) differences by Duncan’s tests. CK, no any additional treatment; compound fertilizer, application of potassium sulfate compound fertilizer; PR1500, application of *P*. *cuspidatum* residues at a rate of 1500 kg/667 m^2^; PR2500, application of *P*. *cuspidatum* residues at a rate of 2500 kg/667 m^2^; PR4000, application of *P*. *cuspidatum* residues at a rate of 4000 kg/667 m^2^.

### Changes in chlorophyll and gas exchange

Except for the PR4000 treatment, which did not affect the SPAD value, the fertilizer, PR1500, and PR2500 treatments all significantly increased the SPAD value by 38.7%, 44.0%, and 54.0%, respectively, compared with the CK treatment (**Table 3**). Regarding leaf gas exchange variables, compared to the CK treatment, the fertilizer treatment significantly enhanced leaf net photosynthetic rate, transpiration rate, intercellular CO_2_ concentration, and stomatal conductance by 18.6%, 34.4%, 11.8%, and 14.2%, respectively. The PR1500 treatment significantly increased leaf net photosynthetic rate, transpiration rate, and intercellular CO_2_ concentration by 14.9%, 26.2%, and 8.1%, respectively. The PR2500 treatment significantly enhanced leaf net photosynthetic rate, transpiration rate, intercellular CO_2_ concentration, and stomatal conductance by 39.1%, 57.9%, 22.0%, and 33.9%, respectively. In contrast, the PR4000 treatment did not significantly alter any of the leaf gas exchange variables.

**Table 3:** Effects of *Polygonum cuspidatum* residues returning to field for two years on leaf chlorophyll and gas exchange variables in three-year-old *P*. *cuspidatum* plants.

Treatments	SPAD value	Net photosynthetic rate (μmol/m^2^/s)	Transpiration rate (mmol/m^2^/s)	Stomatal conductance (mmol/m^2^/s)	Intercellular CO_2_ concentration (μmol/mol)
CK	29.2 ± 2.6 b	5.17 ± 0.23 c	2.36 ± 0.14 c	0.120 ± 0.012 c	278 ± 11 c
Compound fertilizer	40.5 ± 4.2 a	6.13 ± 0.21 b	3.18 ± 0.21 b	0.137 ± 0.003 b	311 ± 9 b
PR1500	42.0 ± 1.4 a	5.94 ± 0.11 b	2.98 ± 0.21 b	0.130 ± 0.006 bc	300 ± 11 b
PR2500	45.0 ± 4.9 a	7.19 ± 0.22 a	3.73 ± 0.11 a	0.160 ± 0.008 a	339 ± 16 a
PR4000	32.2 ± 1.4 b	5.21 ± 0.26 c	2.48 ± 0.24 c	0.124 ± 0.011 c	283 ± 11 c

Different letters following the data (means ± standard error, *n* = 5) from the same column indicated significant (*p* < 0.05) differences by Duncan’s tests. CK, no any additional treatment; compound fertilizer, application of potassium sulfate compound fertilizer; PR1500, application of *P*. *cuspidatum* residues at a rate of 1500 kg/667 m^2^; PR2500, application of *P*. *cuspidatum* residues at a rate of 2500 kg/667 m^2^; PR4000, application of *P*. *cuspidatum* residues at a rate of 4000 kg/667 m^2^.

### Changes in IAA and ZR levels in roots

**Figure 1:**
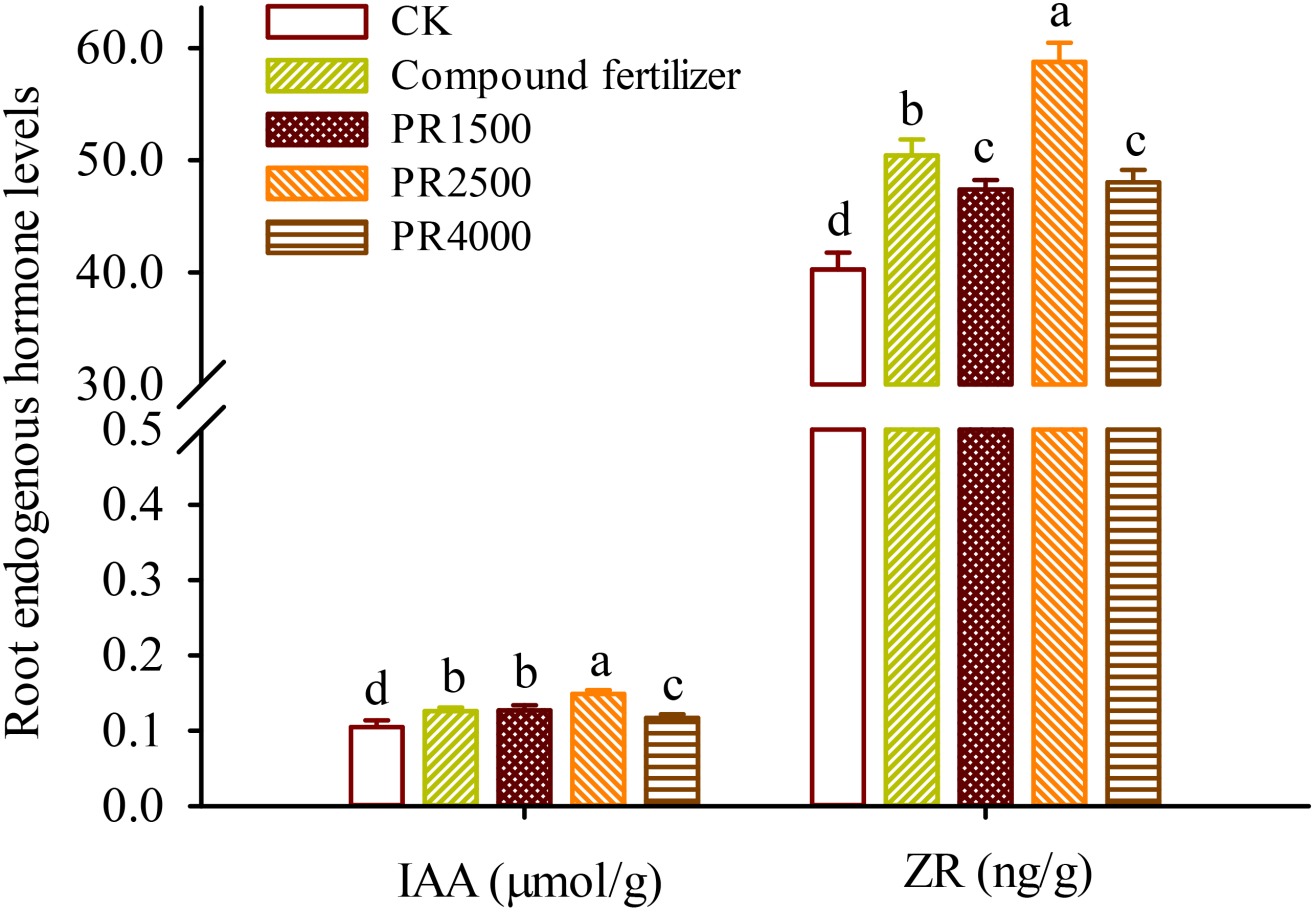
Effects of *Polygonum cuspidatum* residues returning to field for two years on IAA and ZR levels in roots of three-year-old *P*. *cuspidatum* plants. Different letters above the bars (means ± standard error, *n* = 5) indicated significant (*p* < 0.05) differences by Duncan’s tests. CK, no any additional treatment; compound fertilizer, application of potassium sulfate compound fertilizer; PR1500, application of *P*. *cuspidatum* residues at a rate of 1500 kg/667 m^2^; PR2500, application of *P*. *cuspidatum* residues at a rate of 2500 kg/667 m^2^; PR4000, application of *P*. *cuspidatum* residues at a rate of 4000 kg/667 m^2^.

To elucidate the regulatory mechanisms of PR treatments on the growth of *P*. *cuspidatum*, the levels of IAA from auxins and ZR from cytokinins were measured in the roots. Root IAA levels ranged from 0.105 μmol/g to 0.149 μmol/g, and root ZR levels were 40.25−58.76 ng/g (**Figure 1**). Compared with the CK treatment, the fertilizer, PR1500, PR2500, and PR4000 treatments all significantly boosted IAA levels in roots by 19.4%, 20.9%, 41.7%, and 11.0%, respectively, as well as ZR levels in roots by 25.3%, 17.8%, 46.0%, and 19.4%, respectively.

### Changes in active component levels in roots

**Figure 2:**
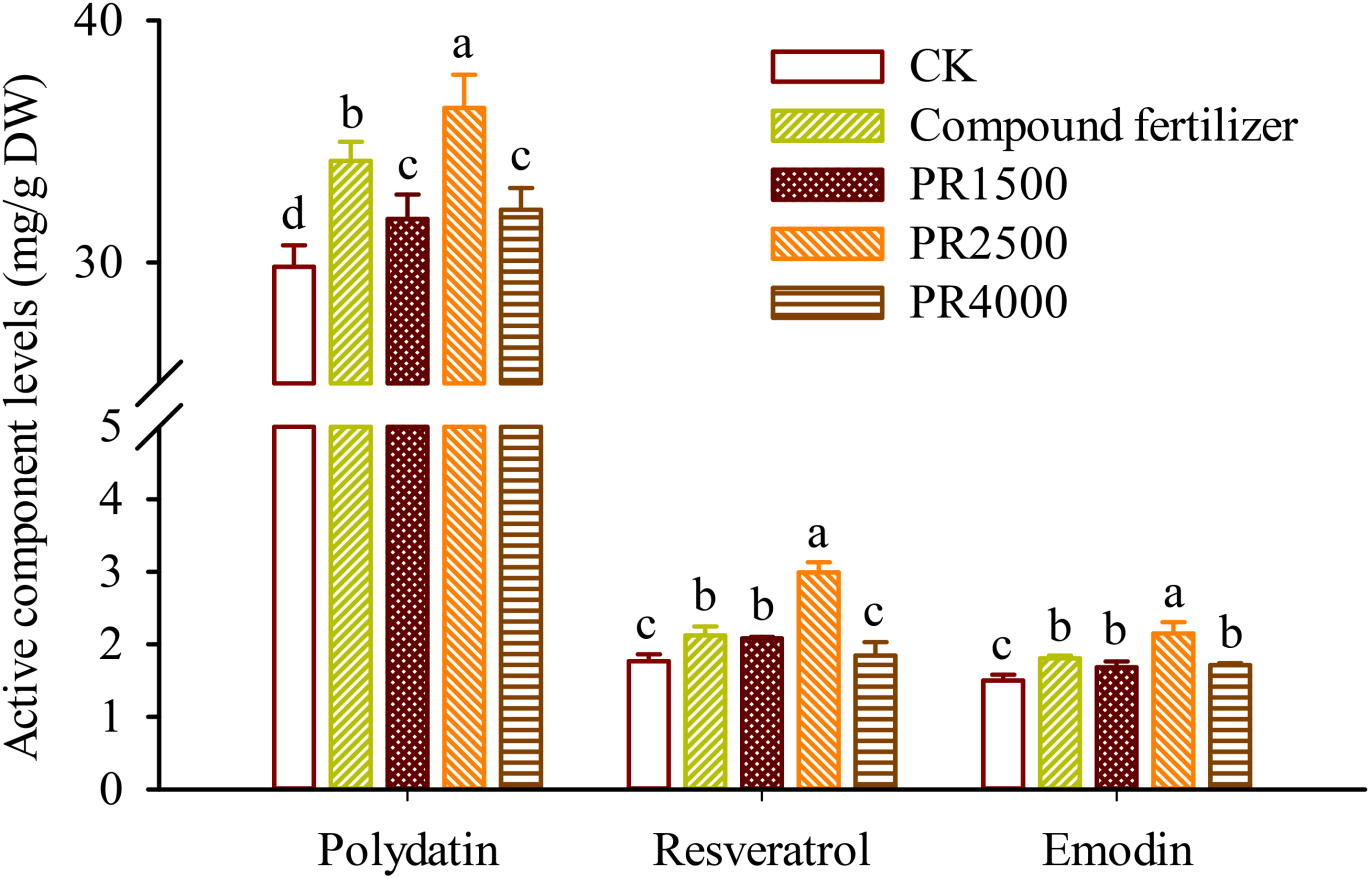
Effects of *Polygonum cuspidatum* residues returning to field for two years on polydatin, resveratrol, and emodin levels in roots of three-year-old *P*. *cuspidatum* plants. Different letters above the bars (means ± standard error, *n* = 5) indicated significant (*p* < 0.05) differences by Duncan’s tests. CK, no any additional treatment; compound fertilizer, application of potassium sulfate compound fertilizer; PR1500, application of *P*. *cuspidatum* residues at a rate of 1500 kg/667 m^2^; PR2500, application of *P*. *cuspidatum* residues at a rate of 2500 kg/667 m^2^; PR4000, application of *P*. *cuspidatum* residues at a rate of 4000 kg/667 m^2^.

High-performance liquid chromatography analysis showed that the roots of *P*. *cuspidatum* contained polydatin at levels of 29.82–36.39 mg/g DW, resveratrol at 1.77–2.99 mg/g DW, and emodin at 1.50–2.15 mg/g DW (**Figure 2**). Compared to the CK treatment, the fertilization and the application of PR1500, PR2500, and PR4000 significantly boosted polydatin levels in roots by 14.7%, 6.6%, 22.0%, and 7.9%, respectively (**Figure 2**). Similarly, these treatments also significantly increased emodin levels in roots by 20.4%, 12.1%, 43.3%, and 14.3%, respectively. Additionally, compared to the CK treatment, the fertilization and the application of PR1500 and PR2500 significantly elevated resveratrol levels in roots by 20.0%, 17.8%, and 69.3%, respectively, while no significant changes in resveratrol levels were observed after the application of PR4000.

### Changes in gene expression levels in roots

**Figure 3:**
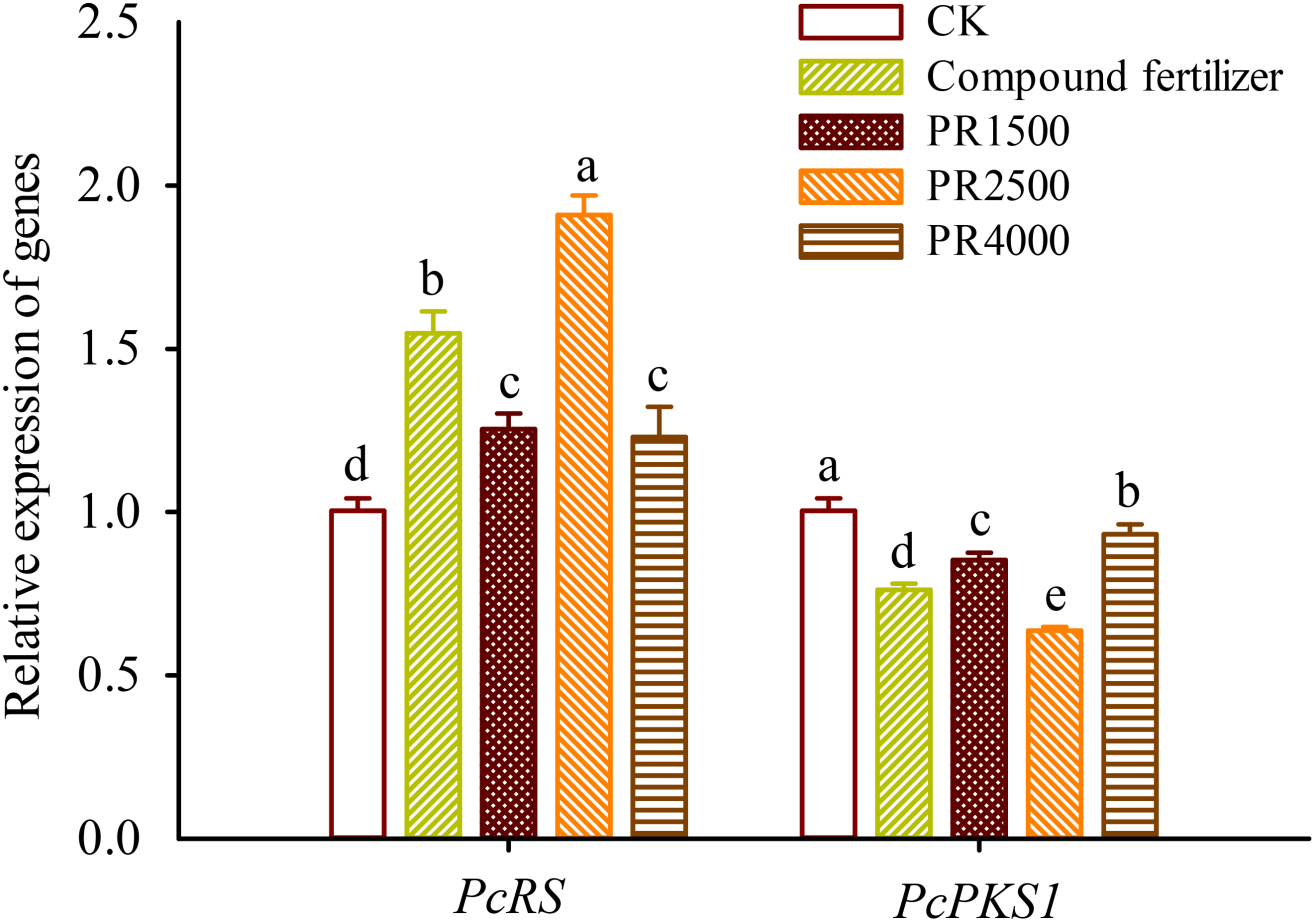
Effects of *Polygonum cuspidatum* residues returning to field for two years on the relative expression of *PcRS* and *PcPKS1* genes in roots of three-year-old *P*. *cuspidatum* plants. Different letters above the bars (means ± standard error, *n* = 5) indicated significant (*p* < 0.05) differences by Duncan’s tests. CK, no any additional treatment; compound fertilizer, application of potassium sulfate compound fertilizer; PR1500, application of *P*. *cuspidatum* residues at a rate of 1500 kg/667 m^2^; PR2500, application of *P*. *cuspidatum* residues at a rate of 2500 kg/667 m^2^; PR4000, application of *P*. *cuspidatum* residues at a rate of 4000 kg/667 m^2^.

Compared to the CK treatment, the fertilization and treatments with PR1500, PR2500, and PR4000 significantly up-regulated the expression levels of the *PcRS* gene in roots by 0.54-, 0.25-, 0.90-, and 0.23-fold, respectively (**Figure 3**). However, the fertilization and treatments with PR1500, PR2500, and PR4000 significantly suppressed the expression levels of the *PcPKS1* gene in roots by 0.24-, 0.15-, 0.37-, and 0.07-fold, respectively.

### Correlationship analysis

**Figure 4:**
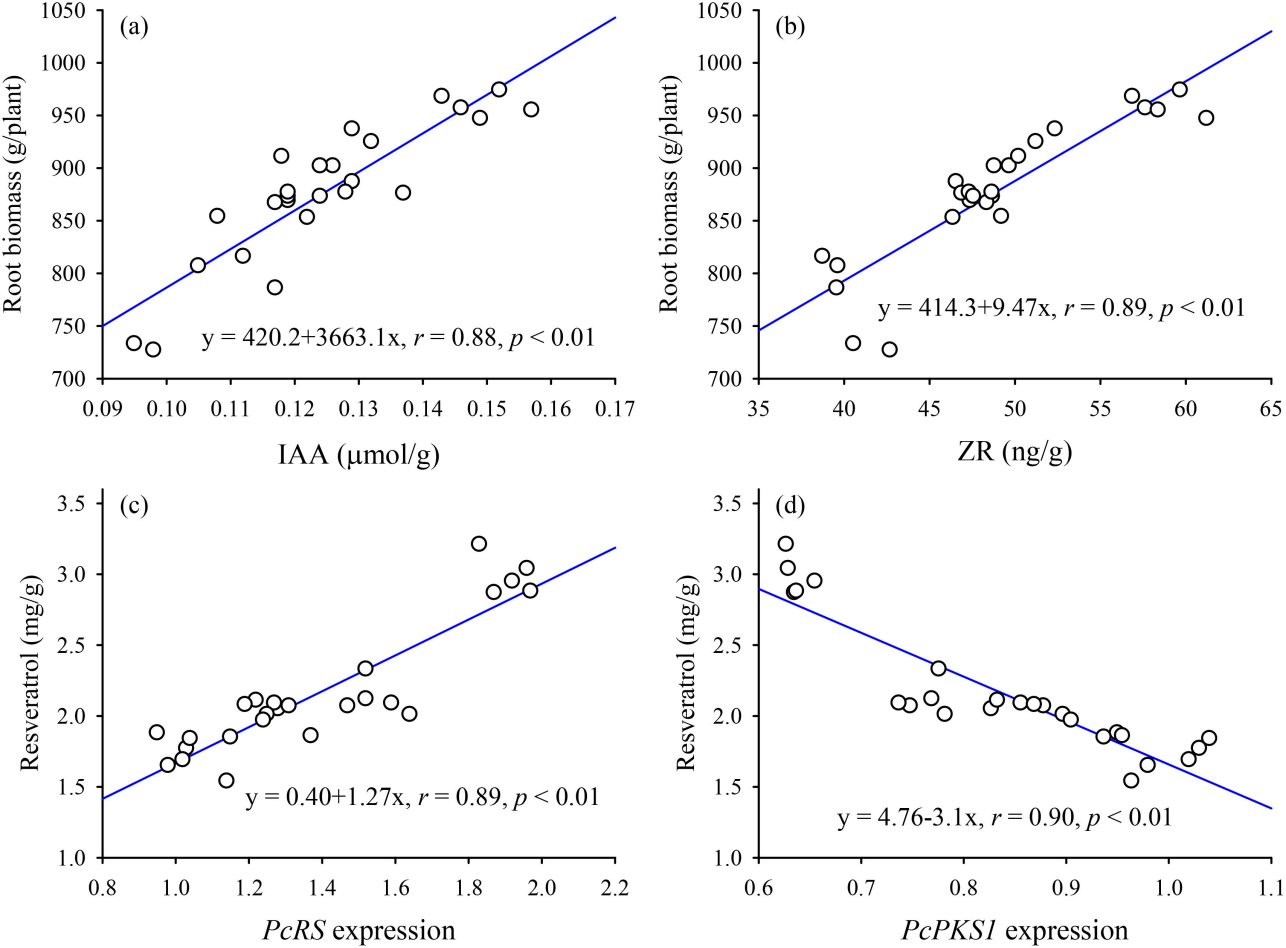
Linear regression between root biomass and root IAA **(a)** and ZR levels **(b)** and between root resveratrol and root *PcRS* **(c)** and *PcPKS1* **(d)** expression (*n* = 25).

Correlation analysis revealed a significantly (*p* < 0.01) positive correlation between root biomass and both root IAA (**Figure 4a**) and ZR (**Figure 4b**) levels. On the other hand, the resveratrol level in roots showed a significantly (*p* < 0.01) positive correlation with the expression of the *PcRS* gene in roots (**Figure 4c**), but a significantly (*p* < 0.01) negative correlation with the expression of the *PcPKS1* gene (**Figure 4d**).

## Discussion

In this study, the application of PR treatments had demonstrated significant enhancements in various plant growth parameters. This finding aligns with previous studies highlighting the positive impact of organic amendments on plant growth and biomass production (Agegnehu et al., 2016; Wan et al., 2024). However, the PR treatments dramatically altered plant growth performance and biomass production, depending on used doses of PRs. Among them, the PR2500 treatment exhibited the superior performance than other PR treatments and compound fertilizer treatment, which could be attributed to the optimal balance of nutrients and organic matter provided by this dose, which likely facilitated robust root development and nutrient uptake (Schulz et al., 2013; Dai et al., 2020). This also suggested that the PR2500 treatment not only enhances aboveground biomass but also significantly promotes root development, which is crucial for overall plant health and resilience. However, the PR4000 (high-dose) treatment also promoted growth, albeit to a lesser extent, indicating that higher doses of PRs may not necessarily induce to proportional increases in plant growth, possibly due to nutrient saturation, potential phytotoxicity from excessive organic matter or secondary metabolites, and allelopathic effects (Zhang et al., 2021).

The SPAD value is a reliable indicator of leaf chlorophyll content, which is directly related to photosynthetic capacity and plant health (Markwell et al., 1995). The PR1500 and PR2500 treatments significantly increased the SPAD value, compared to the CK treatment, suggesting that these treatments effectively enhanced chlorophyll content, potentially improving the plant’s photosynthetic efficiency (Gitelson et al., 2002). In contrast, the PR4000 treatment did not significantly affect the SPAD value, indicating a potential saturation or inhibitory effect at higher application rates, which warrants further investigation.

Leaf gas exchange parameters are critical for assessing plant photosynthetic performance and water-use efficiency (Deng et al., 2024). The low-dose PR (PR1500) treatment also showed significant improvements in the net photosynthetic rate, transpiration rate, and intercellular CO_2_ concentration, and the moderate-dose PR (PR2500) treatment exhibited the most pronounced effects on the net photosynthetic rate, transpiration rate, intercellular CO_2_ concentration, and stomatal conductance. Among all treatments, the PR2500 treatment showed the best results and was significantly superior to the compound fertilizer treatment. This indicated that PR2500 can serve as an alternative to chemical fertilizers for enhancing the growth and improving leaf gas exchange in *P*. *cuspidatum*. The change in gas exchange by PR2500 treatment aligned with the enhanced growth parameters observed in this treatment, indicating a strong correlation between physiological processes and growth outcomes (Dai et al., 2020; Cao et al., 2023). This suggested that a suitable dose of PRs can enhance photosynthetic capacity, possibly through the release of organic matter and nutrients (Chan et al., 2007). Further research is needed to clarify changes in leaf photosynthetic efficiency after PRs treatment through morphological observations (such as stomatal density) and chlorophyll fluorescence parameters. In contrast, the PR4000 treatment did not significantly alter any of the leaf gas exchange variables. This could be due to a potential inhibitory effect at higher application rates, where an excess of nutrients or organic matter may interfere with physiological processes (Zhang et al., 2021).

IAA, a primary auxin, promotes cell division in meristematic tissues, such as root apical meristems, and also influences the differentiation of cells into specialized tissue, such vascular tissues, which plays a role in root development (Woodward and Bartel, 2005; Liu et al., 2023, 2024). ZR, as a cytokinin, promotes cell division by stimulating the transition from the G2 phase to the M phase of the cell cycle, often in coordination with auxins (Mok and Mok, 2001). The PR1500, PR2500, and PR4000 treatments all led to significant increases in root IAA levels, compared to the CK treatment. IAA is a key auxin that promotes cell elongation and is essential for root development and architecture (Roychoudhry and Kepinski, 2022; Rong et al., 2023). The substantial increase in IAA levels, particularly under the PR2500 treatment, suggests that this treatment would boost root growth and development by stimulating auxin biosynthesis or reducing its degradation (Ljung et al., 2005). Similarly, the PR1500, PR2500, and PR4000 treatments also significantly boosted root ZR levels, respectively. ZR is a cytokinin that plays a vital role in cell division, shoot development, and delaying senescence (Sakakibara, 2006). The significant increase in ZR levels, especially under the PR2500 treatment, indicated that this treatment enhanced shoot growth and overall plant vigor by promoting cell division. The application of PR2500 resulted in the most significant increases in both IAA and ZR levels in roots, which aligned with the superior growth performance observed under this treatment. Root IAA and ZR levels were collectively significantly positively correlated with root biomass, suggesting that these hormones enhanced root growth, leading to improved nutrient and water uptake and overall plant vigor. This also suggested that PR amendments can enhance the levels of IAA and ZR in the roots, which synergistically accelerate the production of root biomass. The results underscores the potential of organic amendments like HRs to enhance plant hormonal levels, thereby promoting plant growth and development (Batista-Silva et al., 2024). Emodin, *in vitro*, was found to inhibit the growth of a small amount of soil bacteria and fungal species (Singh et al., 1992), while resveratrol appears to have no significant effect on soil microbial populations (Sohn et al., 2015). Therefore, PRs may indirectly enhance auxin and cytokinin levels by improving soil nutrient availability or modulating enzyme activities in hormone biosynthesis pathways, not microbial activity, which requires further experimental validation. As a result, PRs, especially PR2500, can be a viable alternative to traditional fertilizers, offering both hormonal and growth benefits. In this study, the application of PR treatments significantly affected the levels of key bioactive compounds and resveratrol-associated gene expression in roots of *P*. *cuspidatum* plants. The PR1500, PR2500, and PR4000 treatments led to significant increases in root polydatin levels, respectively, compared to the CK treatment. Polydatin, a resveratrol glucoside, is known for its antioxidant and anti-inflammatory properties (Du et al., 2013). The increase in polydatin levels in roots suggests that these treatments could elevate the production of the active component, which also potentially enhance the plant’s defense mechanisms against oxidative stress and pathogens (Li et al., 2021; Imtiyaz et al., 2024). Similarly, these treatments significantly increased root emodin levels, respectively, with PR2500 showing the most prominent effect. Emodin is a natural anthraquinone with potential therapeutic properties, including anti-inflammatory and anticancer activities (Dong et al., 2016). The substantial increase in emodin levels, particularly under the PR2500 treatment, indicates that this treatment could enhance the plant’s emodin synthesis, potentially leading to improved medicinal properties (Izhaki, 2002). Generally, as the rhizomes of *P*. *cuspidatum* increase in size, the proportion of secondary xylem and secondary phloem also increases (Liu and Hu, 2001). Resveratrol and its glycosides are primarily concentrated in the pith, secondary phloem, and periderm of rhizomes, while emodin is mainly distributed in the secondary xylem of roots (Liu et al., 2015). Since fertilization and various PR treatments significantly increased root biomass, this also implies that the levels of major active components, such as polydatin, emodin, and resveratrol, would increase accordingly.

Resveratrol (3,5,4’-trihydroxystilbene) is a naturally occurring stilbenoid that has attracted significant attention due to its wide range of health benefits and therapeutic potential (Baur and Sinclair, 2006). It is commonly found in various plant species, with *P*. *cuspidatum* being one of the richest sources of this compound (Baur and Sinclair, 2006; Deng et al., 2024). Resveratrol’s potential health benefits have led to its use in dietary supplements, functional foods, and pharmaceuticals (Ke et al., 2023). In the present study, PR1500 and PR2500 treatments significantly elevated root resveratrol levels, respectively, compared to the CK treatment. Moreover, the PR2500 treatment exhibited a significantly greater enhancement in the levels of the three active components in *P*. *cuspidatum* roots than the chemical fertilizer treatment. This strongly suggests that the PR2500 treatment can serve as a promising viable alternative to chemical fertilizers for field production of *P*. *cuspidatum*. Nevertheless, the lack of significant change in resveratrol levels after the application of PR4000 suggested that there may be a complex relationship between the PR4000 treatment and resveratrol metabolism. It could be that PR4000 treatment did not have a significant impact on resveratrol synthesis or accumulation, because excessive application of organic residues could cause microbial imbalance, nutrient immobilization, or root toxicity (Turmel et al., 2015). There was a significantly positive correlation between resveratrol levels and *PcRS* gene expression in roots, which provides a mechanistic insight into the observed increase in resveratrol levels. Since the RS is a key enzyme in the biosynthesis of resveratrol and its glycosylated form polydatin (Jeandet et al., 2010), the up-regulation of *PcRS* gene, especially under the PR2500 treatment, is likely to be directly related to the increased resveratrol and its derivative production. This up-regulation likely drives the metabolic flux towards the stilbene biosynthesis pathway, resulting in higher resveratrol accumulation. The observed increase in resveratrol levels under PR treatments highlights the potential of these strategies to optimize the production of bioactive compounds in *P*. *cuspidatum*, which could have significant implications for the commercial production in *P*. *cuspidatum*.

Nevertheless, the present study also observed that PR1500, PR2500, and PR4000 treatments significantly suppressed the expression levels of the *PcPKS1* gene in roots. It is documented that RS can compete with PKS for the same substrates, *p*-coumaroyl-CoA and malonyl-CoA. In this process, PKS catalyzes the reaction of the butenone-intermediate molecule at the C6 and C1 positions to form naringenin chalcone, while RS catalyzes the reaction of the butenone-intermediate molecule at the C7 and C2 positions to form resveratrol (Liu, 2012). Root resveratrol levels were significantly negatively correlated with root *PcPKS1* expression. The suppression of *PcPKS1* under PRs treatments may indicate a shift in the plant’s metabolic pathways towards the production of resveratrol, potentially at the expense of other secondary metabolites (e.g., naringenin chalcone) (Verpoorte and Memelink, 2002), although this was not demonstrated in this study. The observed suppression of *PcPKS1* and the concomitant increase in resveratrol levels under PR treatments potentially suggest that these interventions redirect metabolic flux towards stilbene biosynthesis. Environmental and biochemical factors can influence the partitioning of metabolic resources in plants (Verpoorte and Memelink, 2002). This underscored the intricate balance between competing biosynthetic pathways in *P*. *cuspidatum*.

## Conclusions

The application of PR2500 emerged as the most effective treatment for enhancing plant growth, biomass production, photosynthetic activities, and root active component levels in *P*. *cuspidatum*. The improvement in root biomass was associated with the elevation of root IAA and ZR levels, and the increase in root resveratrol levels was associated with the up-regulation of root *PcRS* gene expression. An increase in the application rate of PRs from PR2500 to PR4000 led to a reduction in the physiological activity, growth, and active component levels in *P*. *cuspidatum*. It suggests that organic amendments like PRs can be a viable alternative to traditional fertilizers, offering both growth promotion and a shift in metabolic pathways towards the production of beneficial compounds like resveratrol. Therefore, the PR2500 treatment is recommended for the cultivation of *P*. *cuspidatum*. The application of PRs as fertilizer could reduce waste management costs while providing a low-cost organic amendment. In addition, PRs decrease input costs for farmers. However, processing (e.g., composting, pelletizing) and transportation costs of PRs must be factored in for large-scale use. PR’s nutrient content may vary, necessitating standardization. Slow nutrient release of PRs may not meet high-demand crop needs without supplementation. Long-term field studies are needed to assess whether PR application leads to cumulative benefits or potential negative consequences such as nutrient imbalances, soil microbial communities, and organic matter accumulation.

## Data Availability

The original contributions presented in the study are included in the article/**Supplementary Material**. Further inquiries can be directed to the corresponding author.
