# Supplementary material for: Soil organic amendments with Polygonum cuspidatum residues enhance growth, leaf gas exchange, and bioactive component levels
Source: Front Plant Sci. 2025 Apr 25;16:1594905. doi: 10.3389/fpls.2025.1594905 (PMC12061903; doi:10.3389/fpls.2025.1594905)
Supplement: Supplementary file 1 [file Table1.docx]

**Supplementary Table S1** Primer sequences of the genes used in this study for qRT-PCR

| **Genes** | **Gene ID** | **Forward sequence (5’→3’)** | **Reverse sequence (5’→3’)** |
| --- | --- | --- | --- |
| *PcActin* | MK288156.1 | TACAATGAGCTTCGGGTTGC | GCTCTTTGCAGTTTCCAGCT |
| *PcPKS1* | EF090604.1 | GTACACAACAGAGAGATAACTGTC | GGTCATGTGCTCGCTGTTGGTG |
| *PcRS* | DQ900615. 1 | GAGATGACGAAGGCACTAACA | GGAAGTAGAAGTCGGGAAAGTC |
